# New insights into the structural and spatial variability of cell-wall polysaccharides during wheat grain development, as revealed through MALDI mass spectrometry imaging

**DOI:** 10.1093/jxb/eru065

**Published:** 2014-03-05

**Authors:** Dušan Veličković, David Ropartz, Fabienne Guillon, Luc Saulnier, Hélène Rogniaux

**Affiliations:** INRA, UR1268 Biopolymers Interactions Assemblies F-44316 NANTES, France

**Keywords:** Acetylated arabinoxylan, beta glucans, cell wall, imaging, in-situ digestion, mass spectrometry, wheat.

## Abstract

Arabinoxylans (AX) and (1→3),(1→4)-β-glucans (BG) are the major components of wheat grain cell walls. Although incompletely described at the molecular level, it is known that the chemical and distributional heterogeneity of these compounds impacts the quality and use of wheat. In this work, an emerging technique based on MALDI mass spectrometry imaging (MSI) was employed to map variations in the quantity, localization, and structure of these polysaccharides in the endosperm during wheat maturation. MALDI MSI couples detailed structural information with the spatial localization observed at the micrometer scale. The enzymic hydrolysis of AX and BG was performed directly on the grain sections, resulting in the efficient formation of smaller oligosaccharides that are easily measurable through MS, with no relocation across the grain. The relative quantification of the generated oligosaccharides was achieved. The method was validated by confirming data previously obtained using other analytical techniques. Furthermore, *in situ* analysis of grain cell walls through MSI revealed previously undetectable intense acetylation of AX in young compared to mature grains, together with findings concerning the feruloylation of AX and different structural features of BG. These results provide new insights into the physiological roles of these polysaccharides in cell walls and the specificity of the hydrolytic enzymes involved.

## Introduction

**Fig. 1.**
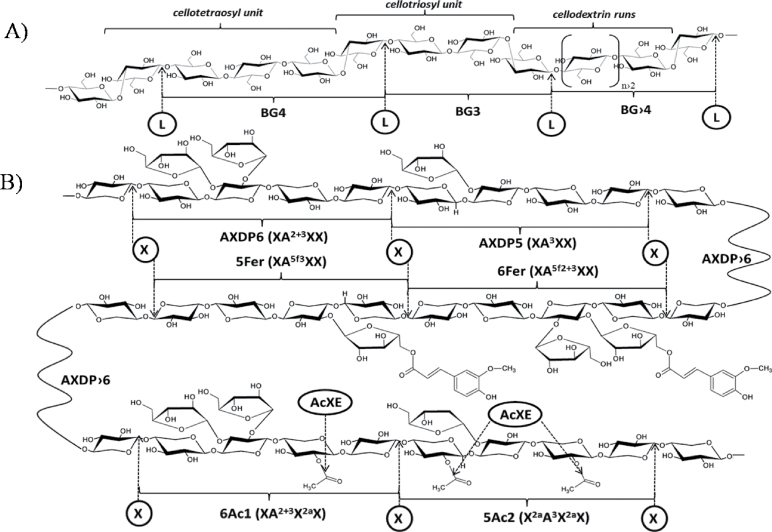
Structure of β-glucans (A) and arabinoxylans (B). Bonds that are susceptible to hydrolysis by the enzymes used in this study (L, lichenase; X, xylanase; AcXE, acetylxylan esterase) are marked with arrows, and main oligomers released by enzymic digestion are labelled as in the manuscript. Arabinoxylan (AX) oligomers are also abbreviated according to the Fauré *et al*. (2009) nomenclature for heteroxylans (in parentheses): starting position is nonreducing d-xylosyl unit; X, unsubstituted xylosyl residue; A, xylosyl residue substituted by l-arabinofuranosyl residue; a, acetyl residue; f, feruloyl residue; 2, (1→2) linkage; 3, (1→3) linkage; 5, (1→5) linkage. Note that lichenase specifically cleaves the β (1→4) linkage of 3-*O*-substituted d- glucopyranosyl residue in β-glucans (BG) and xylanase specifically attacks β (1→4) glycosidic bonds between two unsubstituted xylopyranosyl residues in AX.

Although the polysaccharides found in wheat grain cell walls account only for 2–4% of dry weight, these compounds significantly affect the end uses of wheat grain, such as for milling, baking, brewing, and animal feeding, due to their viscosity and hydration properties in aqueous solution (Fincher and Stone, 1986; Saulnier *et al.*, 2007*a*). As a dietary fibre, polysaccharides also have a major influence on the nutritional quality of wheat grain (Fincher and Stone, 1986). The two main components of the cell wall of wheat grain endosperm are arabinoxylans (AX) and mixed-linkage β-glucans (BG) (Saulnier *et al.*, 2012), which structures are depicted in Fig. 1. Arabinoxylans are β-(1→ 4) d-xylan polymers in which d-xylopyranosyl residues can be mono-substituted at the *O*3 position or di-substituted at the *O*2 and *O*3 positions with α-l-arabinofuranosyl residues. Additional modifications of AX, such as acetylation of the xylan backbone and feruloylation of arabinofuranosyl residues, have also been observed in wheat grain (Saulnier *et al.*, 2007*a*, *b*). Mixed-linkage β-glucans consist of a linear chain of β-d-glucopyranosyl residues linked by (1→4) and (1→3) glucosidic linkages. These molecules can be roughly described as copolymers of cellotriosyl and cellotetraosyl units with a small proportion of cellodextrin runs. The ratio between these units can be considered a ‘fingerprint’ of the BG structures in cereals, which strongly influences the physicochemical properties of BG and the consequent health benefits of these cereals as dietary fibres (Lazaridou and Biliaderis, 2007). Subtle, albeit significant, differences in AX and BG structures are expressed during the development of wheat grain endosperm and among different tissues within a given wheat grain (Philippe *et al.*, 2006*a*–*c*; Saulnier *et al.*, 2009; Toole *et al.*, 2010; Robert *et al.*, 2011).

Because the structural variability of AX and BG plays a significant role in the properties of cell walls, potentially impacting the development and processing quality of wheat grains, obtaining adequate insight into the occurrence and spatial distribution of these biopolymers across the grain is of considerable interest. This work should couple chemical information with spatial information, which has previously been proposed through imaging coupled with antibody labelling (Philippe *et al.*, 2006*a*), FT-IR microspectroscopy (Philippe *et al.*, 2006*b*; Saulnier *et al.*, 2009; Robert *et al.*, 2011; Toole *et al.*, 2010, 2011), or Raman microspectroscopy analyses (Philippe *et al.*, 2006*c*; Toole *et al.*, 2009; Robert *et al.*, 2011). Microdissection of tissue coupled with enzymic fingerprinting analysis has also been reported (Saulnier *et al.*, 2009). These methods are time consuming because of the tedious sample preparation and/or long acquisition times as well as heavy data processing (vibrational spectroscopy) required, and cannot be envisaged for use in a large series of samples. There are many imaging techniques with a high resolution (e.g. transmission electron microscopy, fluorescence microscopy, atomic force microscopy) but unless an appropriate antibody or tagging reagent is developed, these approaches are not efficient in revealing chemical changes in polysaccharide cell-wall structures.

Matrix-assisted laser desorption/ionization (MALDI) mass spectrometry imaging (MSI) has recently emerged as a powerful method for resolving both the spatial distribution and structures of many kinds of molecules in intact tissue sections. Following tissue mounting on a conductive glass plate and application of the MALDI matrix, the MS instrument captures a series of mass spectra, each of which represents the mass proﬁle of a laser beam-irradiated region of the sample. The major advantage of MSI over other imaging techniques is that various compounds present at the surface of a tissue can be mapped without the need for making prior assumptions about which molecules are likely to be present. As a consequence, molecular structures and their potential modifications can be monitored simultaneously, and a large volume of informative data can be generated without excessive investment in sample preparation (Kaspar *et al.*, 2011). Due to the heterogeneity of MALDI matrix crystal distributions and inhomogeneity of microenvironments (e.g. produced by salt or pH gradients, background structures, or neighbouring molecules), the intensity of mass spectra might be affected, and significant information could be lost in the created images (Deininger *et al.*, 2011). Hence, introducing an appropriate internal standard (Källback *et al.*, 2012), the application of a normalization process (Deininger *et al.*, 2011) and improvement of matrix selection and application (Goodwin, 2012) are necessary steps for obtaining realistic, quantitative images. MALDI MSI has been successfully employed to map small metabolites, lipids, drugs, peptides, or small proteins for diverse purposes in the context of clinical investigations (for a recent review, see Angel and Caprioli, 2013; Nimesh *et al.*, 2013). However, little emphasis has been given to the applications of MSI in plant biology (Kaspar *et al.*, 2011), although increased knowledge regarding the localization and dynamics of specific molecules is essential to elucidate physiological processes. In addition, most of the reports describing the application of MSI in plants have focused on lipid metabolites (Horn *et al.*, 2012; Lee *et al.*, 2012). Investigations of carbohydrate derivatives, such as glucosinolates (Shroff *et al.*, 2008) and flavonoid glucosides (Perdian and Lee, 2010; Franceschi *et al.*, 2012) and particularly pure sucrose (Burrell *et al.*, 2007; Ye *et al.*, 2013) or water-soluble carbohydrates (Robinson *et al.*, 2007; Lunsford *et al.*, 2011), are scarce. As far as is known, none of these MSI studies have focused on the imaging of complex cell-wall polysaccharides, despite the asymmetric distribution of these compounds throughout plant tissues (Harris and Smith, 2006) and the important roles of these molecules regarding cell-wall properties, plant tissue cohesion, organ development, and defence processes (Vorwerk *et al.*, 2004).

In this context, this study employed MSI to analyse the variability of the abundance, structure and distribution of AX and BG in the endosperm (starchy endosperm and aleurone layer) of developing wheat (*Triticum aestivum*) grains. This work took advantage of an enzymic fingerprinting technique that was previously developed in this study group’s laboratory to screen for structural variations in AX and BG in cereal grains (Saulnier *et al.*, 2009). The approach applied in the present study involved the *in situ* enzymic hydrolysis of endosperm polysaccharides, followed by analysis of the spatial distribution of the generated oligosaccharides using MALDI mass spectrometry. The results provide new insights into the structure-function relationships of these polymers in wheat grain.

## Materials and methods

### Biological materials

Winter soft wheat, *Triticum aestivum* L. cv. Recital, was grown in a glasshouse in pots under conditions of natural day length at the INRA Station of Le Rheu (France). Grains were collected at 245 D (referred to as the ‘young’ stage) and 700 D (referred to as the ‘mature’ stage), as previously described (Philippe *et al.*, 2006*a*). After harvesting, the mature grains were stored at 4 °C, whereas the young grains were transferred to 70% ethanol for fixation, and the samples were subsequently stored at 4 °C.

### Chemicals and reagents

Maltotriose (MT), 2,5-dihydroxybenzoic acid (DHB), and aniline were purchased from Sigma-Aldrich (Saint Quentin Fallavier, France). *N*,*N*-Dimethylaniline (DMA) was obtained from Fisher Bioblock Scientific (Illkirch, France). Xyloglucan heptasaccharide (XXXG; Supplementary Fig. S1 available at *JXB* online), which was used as an internal standard, was procured from Megazyme (Bray, Ireland). Purified galactomannan digests (degree of polymerisation, DP, 3–9), which were used as mass calibration standards for MALDI-TOF, were kindly provided by the Laboratoire de Chimie des Substances Naturelles (EA 1069, Université de Limoges, France). Acetonitrile (ACN), ethanol (EtOH), and methanol (MeOH) were of HPLC grade (Carlo-Erba Reagents, Val de Reuil, France). Water of ultrapure quality was obtained using Milli-Q apparatus (Millipore, Molsheim, France).

### Enzymes

Endoxylanase (endo-1,4-β-d-xylanase; EC 3.2.1.8, CAZy GH family 11; http://www.cazy.org/) from *Trichoderma viride* was purchased from Megazyme (xylanase M1, Bray, Ireland). The specific activity of the enzyme preparation determined by the supplier on water-extractable arabinoxylans (WE-AX) (40 °C, pH 4.5) was 2300U ml^–1^ and the optimum pH was 4.5–5. Lichenase (endo-1,3(4)- β-d-glucanase; EC 3.2.1.73, CAZy GH family 16) from *Bacillus subtilis* was obtained from Megazyme. The specific activity of the enzyme preparation determined by the supplier on barley β-glucan (40 °C, pH 6.5) was 1000U ml^–1^ and the optimum pH was 6.5–7.0. Acetylxylan esterase (EC 3.1.1.72, CAZy CE family 6) from *Orpinomyces* sp. was purchased from Megazyme. The specific activity of the enzyme preparation determined by the supplier on *p*-nitrophenyl acetate (40 °C, pH 6.7) was 1000U ml^–1^ and the optimum pH was 7.0. The enzymes were desalted just before use with a PD-10 column (GE Healthcare) using distilled water.

### Sample preparation for MALDI-MS imaging

The tissues were prepared according to Philippe *et al.* (2006*b*), with slight modifications. In the mature stage of wheat development, the embryo was excised and the grain was water-soaked between filter paper moistened with distilled water (16h at 4 °C). Because the young wheat grains were naturally hydrated, these samples did not require soaking. Sectioning was performed using a Vibratome (Microm Microtech, Francheville, France) in 70% ethanol. Transverse sections (60 μm) were stored in 70% ethanol at 4 °C until further analysis. Just before use, the tissue sections were washed with 50% ethanol and then consecutively washed with water to remove small oligosaccharides (DP3–DP6), which are present in the tissue and overlap with the BG signals.

After washing, without any other additional treatment, the sections were mounted on indium tin oxide (ITO) glass slides (cat. no. 237001, Bruker Daltonik, Bremen, Germany) using conductive carbon tape as a support.

### *In situ* digestion of cell-wall polysaccharides

Enzymic digestion of samples was performed using 2U ml^–1^ lichenase and 4.6U ml^–1^ xylanase. The enzymes were homogeneously applied as fine droplets at the tissue surface using an in-house-designed spraying robot. Briefly, this procedure involved adapting an electrospray probe dismounted from an LCQ Advantage (Thermo-Fisher Scientific) to an X,Y,Z robotic arm (F4300N purchased from FISNAR). Spraying of the liquid was achieved by connecting the electrospray probe to a syringe pump (flow rate of 600 μl h^–1^) and assisted pneumatically with nitrogen (1×10^5^ Pa). This robot enables high reproducibility of enzyme application due to the well-controlled process of the consumption and deposition of the liquid. In all experiments, the distance between the needle tip of the electrospray probe and the tissues was 3cm (Z-axis), and a brush rectangle pattern of robot head movement (distance between lines 1mm) was used, with each cycle being performed along the opposite axis (X or Y) as the previous cycle. The movement speed of the robot head was 5mm s^–1^. Other parameters (X and Y axis start and end coordinates, volume of the enzyme, number of cycles) were set to ensure that the robot consistently deposited 0.3 μl of enzyme per mm^2^ of sprayed area (corresponding to 0.0014U xylanase and 0.0006U lichenase per mm^2^ tissue). After spraying, the tissues were transferred to a closed container with saturating humidity to maintain some relative humidity during incubation with the enzymes. The samples were incubated at 40 °C for 3h since further incubation (up to 16h) did not make any significant difference in qualitative and quantitative imaging results.

### Digestion of cell-wall polysaccharides

Water-soluble cell-wall polysaccharides were isolated from a water extract of wheat flour as previously described (Dervilly *et al.*, 2000). This mixture contained AX and BG at ratio 80:20 (w/w), similar to that found in endosperm cell walls, and was used as a control for the end products of xylanase and lichenase action. The mixture of polysaccharides (5mg) was dissolved in water (0.8ml) and transferred to an Eppendorf tube, to which 0.2ml of an enzyme solution containing 1U lichenase and 1U xylanase was added, followed by incubation at 40 °C for 16h. The supernatant was subsequently placed in a boiling water bath for 10min, filtered through a 0.45-μm filter and frozen. The sample was diluted 10 times prior to MALDI-TOF MS analysis.

### Relative quantification of released oligosaccharides: application of an internal standard

Xyloglucan heptasaccharide (XXXG) was used as an internal standard for normalization and relative quantification. Uniform application was performed using the same apparatus and conditions as applied for the enzymes (see above). The following amounts of XXXG were deposited: 0.076 μg (mm_tissue_)^–2^ for comparisons between mature and young wheat; 0.076 μg (mm_tissue_)^–2^ for comparisons in young wheat; and 0.76 μg (mm_tissue_)^–2^ for comparisons in mature wheat. The construction of calibration curves for testing the linearity of the response was performed after applying 1 μl MT (concentrations ranging from 0 to 100 μg ml^–1^) and two known concentrations of XXXG (0.02 or 2 μg mm^–2^) on the ITO target plate. The estimated contact area between a 1-μl droplet and the ITO plate was 2mm^2^.

To compare the quantities of BG and AX in different stages of development (mature versus young, m vs. y), the following equations were used, assuming that the selected oligomers are the main end products of enzymic digestion (Ordaz-Ortiz *et al.*, 2005; Saulnier *et al.*, 2009):

$$\frac{BG_{m}}{BG_{y}}=\frac{\frac{{\left(BG3+BG4\right)}_{m}}{XXXG_{m}}}{\frac{{\left(BG3+BG4\right)}_{y}}{XXXG_{y}}}\text{ and }\frac{AX_{m}}{AX_{y}}=\frac{\frac{{\left(AX5+AX6+AX7\right)}_{m}}{XXXG_{m}}}{\frac{{\left(AX5+AX6+AX7\right)}_{y}}{XXXG_{y}}}$$

One critical assumption is that the ionization efficiency of all DPs released from a given polysaccharide is similar. Although this might not be true for all oligosaccharides, there is convincing evidence that, for the examined DPs, there is not a great deal of variation in the ionization efficiency (Harvey, 1993; Choi and Ha, 2006).

### MALDI matrices

An ionic DHB/DMA matrix that was previously demonstrated to be suitable for the detection of many oligosaccharides (Ropartz *et al.*, 2011) was used in this study. The matrix was prepared as an equimolar mixture of DHB and DMA (DHB 100mg ml^–1^ in H_2_O/ACN/DMA, 1:1:0.02). Using this MALDI matrix, only sodium adducts of the studied species were detected ([M+Na]^+^).

In addition, an aniline/DHB matrix was used to ascertain the origin of the acetylated ions. This matrix was prepared according to Snovida *et al.* (2006) (DHB 100mg ml^–1^ in 1ml H_2_O/ACN/aniline, 1:0.02).

### Application of the MALDI matrix for MSI

Application of the MALDI matrix was performed using an automatic vibration vaporization system, (ImagePrep, Bruker Daltonik). The system settings were as follows: 52 cycles; 25% spray power; 30% spray modulation; 2 s spray time; 30 s incubation time; and 60 s dry time (N_2_ flow provided at 2×10^5^ Pa).

### MSI analysis

All MSI measurements were performed in an Autoflex III MALDI-TOF/TOF spectrometer (Bruker Daltonik) equipped with a Smartbeam laser (355nm, 200 Hz) and controlled using the Flex Control 3.0 software package. The mass spectrometer was operated with positive polarity in reflectron mode, and spectra were acquired in the range of 500–2000 m/z.

The laser raster size was set at 100 μm, which is approximately equal to the laser spot diameter. At this resolution, it takes approximately 30min to complete an image of one wheat section. The signal was initially optimized by manually adjusting the laser power and the number of laser shots fired. According to this procedure, full-scan MS experiments were run with 200 laser shots per step and using the laser power that generated the best signal-to-noise ratio. Image acquisition at tissue surfaces was performed using Flex Imaging 2.1 software (Bruker Daltonik). Relative comparisons of the released oligosaccharides in the different tissue sections and of the ratios of different ions in the same tissue section were performed through labelled normalization using MALDI Tools 1.1 software (Källback *et al.*, 2012) compatible with Flex Imaging 2.1.

## Results

### *In situ* enzymic digestion of cell-wall polysaccharides

Intact AX and BG polymers cannot be directly analysed through MALDI MS due to their high molecular masses (200–500kDa), heterogeneity, and the fact that these compounds are tightly embedded within cell walls. Thus, these polymers must be enzymically hydrolysed to generate smaller oligosaccharides that can be efficiently extracted from the MALDI matrix layer and detected. To reduce any bias in the obtained BG and AX composition due to an incomplete enzymic action, hydrolysis was performed to generate end products (Wood *et al.*, 1991; Ordaz-Ortiz *et al.*, 2005). The conditions for the enzymic degradation of AX and BG have been described in samples of flours and ground grains of cereals, and the end products have been thoroughly identified (Ordaz *et al.*, 2005; Saulnier *et al.*, 2009). As shown in Fig. 1, the action of *B. subtilis* lichenase on BG mainly produces gluco-oligosaccharides (GOS) of DP3 and 4 (cellobiosyl-glucose and cellotriosyl-glucose, BG3 and BG4, respectively) as well as small proportions of GOS of higher DPs (Ghotra *et al.*, 2008; Saulnier *et al.*, 2009). Depending on the arabinosyl substitution pattern of the xylan chain involved, *T. viride* endoxylanase releases xylose, xylobiose, and various arabino-xylo-oligosaccharides (AXOS), with degree of polymerization from 4 to 30 (http://jcggdb.jp/GlycoPOD/protocolShow.action?nodeId=t217; Saulnier *et al.*, 2009; Fig. 1).

To avoid any delocalization of the oligosaccharides formed after hydrolysis, a limited amount of water was used during the *in situ* digestion step performed in the MALDI MSI experiments, thereby restricting the efficiency of enzymic action. The products released following the *in situ* enzymic digestion of AX and BG in the wheat grain sections were, thus, compared to those formed after full digestion of isolated water-soluble extracts of wheat flour containing AX and BG (Supplementary Fig. S2 available at *JXB* online). The spectral profile of the released oligosaccharides was the same in the two experiments, showing that conditions for complete *in situ* digestion were achieved.

**Fig. 2.**
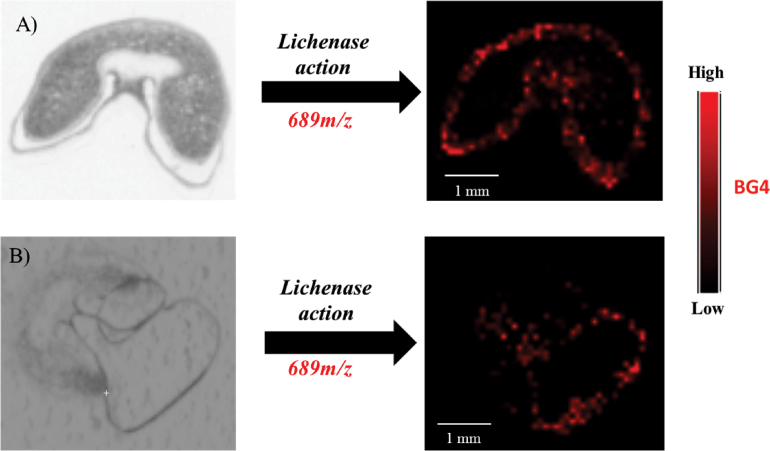
Distribution of ions at 689 m/z, assigned to the β-glucan 4 (BG4) signal in native young wheat (A) and after displacing the outer layer from young wheat sections (B).

Next, this work assessed whether any significant diffusion (i.e. considering the 100-μm resolution of the acquired MALDI MSI images) of the compounds occurred under these conditions. In this analysis, the outer layer (most probably consisting of the pericarp) of a young wheat grain section was carefully displaced from its original position using tweezers, prior to the application of lichenase (Fig. 2). In the intact/native grain section, the signal of DP4 GOS (BG4) released from BG is detected on the border of the grain. When the outer layer is displaced from its natural position, the BG4 signal is accordingly moved (Fig. 2B). This result confirms that this distribution does not reflect uncontrolled diffusion of the molecules from the central to the peripheral cells of the endosperm or the fact that the tissue-suppressive effect (i.e. a decrease of the signal intensity due to the interference of the tissue) could be lower in this region due to the surrounding out-of-tissue (OT) area.

### Application of the MALDI matrix and relative quantification

Based on previous results (Ropartz *et al.*, 2011), a MALDI matrix comprised of an equimolar mixture of DHB and DMA was selected to detect the oligosaccharides released following the enzymic degradation of AX and BG in wheat endosperm. An important issue of this study was the comparison of the relative abundances of selected molecular structures across the grain and/or among tissues (e.g. to compare developmental stages). However, the signal intensity obtained in MALDI mass spectra depends on the number of laser shots and the amount and properties of the deposited matrix (Snovida and Perreault, 2007). Moreover, in MSI experiments, the signal intensity can be altered due to the interference of tissues: different tissues or distinct areas of the same tissue might exhibit different responses for the same quantity of a compound (Stoeckli *et al.*, 2007; Källback *et al.*, 2012). Thus, careful control of the signal intensity and signal normalization must be achieved to allow quantitative comparison of tissues (Deininger *et al.*, 2011). The use of an internal standard, introduced as a known quantity in samples, is common for this purpose (Källback *et al.*, 2012). The internal standard should exhibit a behaviour close to that of the investigated compound but not interfere with the detection of that compound. Ideally, an isotope analogue is used for this purpose. However, in the present study, the production and reliable deposition of isotopically labelled analogues of the investigated molecules throughout the tissues was not readily achievable. Thus, an oligosaccharide derived from xyloglucan showing a degree of polymerization of seven was chosen as an internal standard: this oligosaccharide consists of a cellotetraose substituted with three α-d-xylopyranosyl residues at position six of the first, second, and third glucopyranosyl residues from the nonreducing end (XXXG, *M*_r_=1062g/mol) (Supplementary Fig. S1 available at *JXB* online). This compound is affordable as a pure material, and as a neutral oligosaccharide, it displays a behaviour similar to AXOS and GOS in MALDI MS. The presence of this standard should not mask the detection of the investigated molecules, as its mass does not overlap with the oligohexoses released from BG (*M*_r_=162.N + 18) or the oligopentoses released from AX (*M*_r_=132.N + 18). In addition, due to its structure, XXXG cannot be degraded by xylanase or lichenase.

To validate XXXG as an internal standard, MT, which was selected to mimic GOS derived from BG with a degree of polymerization of 3 (BG3), was applied in a controlled quantity to the wheat grain sections. The signal intensity was measured relative to that of XXXG in different regions of interest (ROIs) of young and mature tissues as well as OT (i.e. on an ITO plate not covered with grain tissue; Table 1). When absolute signal intensities were considered, young tissues exhibited much greater amounts of MT and XXXG than mature tissues, although the same quantity was deposited in both stages. Much higher intensities were measured OT. These results clearly highlight the existence of pronounced and heterogeneous interference in the ionization process, associated with the chemical environment of the analysed molecules (Sugiura and Setou, 2010; Hamm *et al.*, 2012). However, when considering the normalized signal, a constant MT/XXXG ratio was obtained for the ROIs of the young and mature grain sections and the OT region.

**Table 1.** MS signal intensity of maltotriose and xyloglucan heptasaccharideData were collected in the same experiment for young and mature grain sections and in three different experiments for a mature wheat section. MT, maltotriose; OT, out of tissue; ROI, different regions of interest; XXXG, xyloglucan heptasaccharide.

Molecule	Set 1				Set 2			
	Applied quantity (ng mm^–2^)	OT	Young	Mature	Applied quantity (ng mm^–2^)	I	II	III
MT	380	47	11	1.3	76	0.22	1.25	0.72
XXXG	76	40	9.5	1.1	76	0.85	4.85	2.9
MT/XXXG ratio	5	1.17	1.16	1.18	1	0.26	0.26	0.25

In a subsequent experiment, the same amount of XXXG and MT was deposited on three mature wheat grain sections (I, II and III), analysed at different times. The signal intensities recorded for both XXXG and MT varied greatly among the three experiments (Table 1), with the variation primarily reflecting the deposition of the MALDI matrix layer, which showed differences in thickness and homogeneity. Yet, as expected, the MT/XXXG ratio remained constant.

Importantly, the intensity of the examined compound (MT) relative to the internal standard (XXXG) was linear (*r* ^2^>0.98) over a wide range of concentrations (Supplementary Fig. S3 available at *JXB* online). However, as shown in these plots, when the concentration of the internal standard was increased from 0.02 to 0.2 μg mm^–2^, the slope of the line did not decrease proportionally. This result suggests that the same concentration of the internal standard must be used between related experiments to allow a reliable comparison. Control and reproducibility of the application of the internal standard were successfully achieved using an in-house-designed spraying robot. Thus, the conditions required for efficient normalization were achieved, making it possible to compare the results in the subsequent experiments, independent of the time of analysis as well as slight variations in MALDI matrix deposition and instrument settings.

### Time-course distribution of AX and BG during wheat grain development

**Fig. 3.**
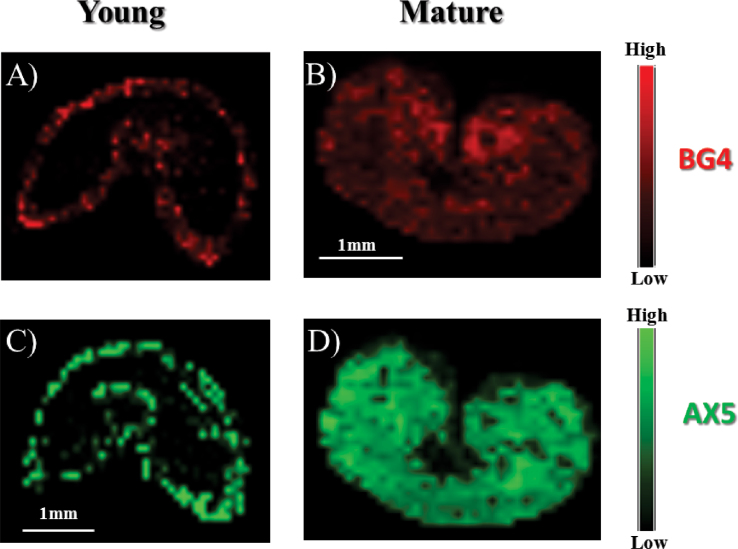
Distribution of β-glucan 4 (BG4, ion at 689 m/z; A and B) and arabinoxylan 5 (AX5, ion at 701 m/z; C and D) released following the on-tissue enzymic digestion of young (A and C) and mature (B and D) wheat sections. Note that the scales indicate the relative signal intensity within a section but cannot be used to compare the intensities between different sections.

The relevance of the information retrieved using the optimized MALDI MSI method was evaluated by investigating young versus mature grains. Much is known about the distribution of the main cell-wall polysaccharides in wheat grain at different stages of development (Philippe *et al.*, 2006*a*–*c*, 2007), and this material can therefore be used as a control. Fig. 3 shows the distribution, in young and mature endosperm tissues, of two of the main oligosaccharides produced by the action of lichenase and endoxylanase, which are DP4 GOS (BG4) and DP5 AXOS (AX5), respectively. In the young stage, BG4 and AX5 were predominantly located in the outer layer, while in the mature stage, it was clear that the starchy endosperm cell walls became evenly enriched in BG and AX, and a uniform distribution of BG4 and AX5 was observed throughout the tissue. These findings are consistent with those of Philippe *et al.* (2006*a*, *b*) obtained using other analytical techniques. In addition, following normalization of the signal relative to XXXG, the current work observed that the overall amount of BG increased 4-fold during maturation, whereas the quantity of AX increased 50-fold. These results are consistent with current knowledge regarding AX and BG accumulation in wheat cell walls, where BG biosynthesis and deposition begins early during seed development and AX is predominantly found in mature wheat grains (Philippe *et al.*, 2006b; Toole *et al.*, 2010). Thus, the appropriateness of MALDI MSI for assessing both the qualitative and quantitative characteristics of the main polysaccharides in the wheat cell wall was successfully demonstrated.

### Structural variations of BG and AX as revealed upon grain maturation by MALDI MSI

**Fig. 4.**
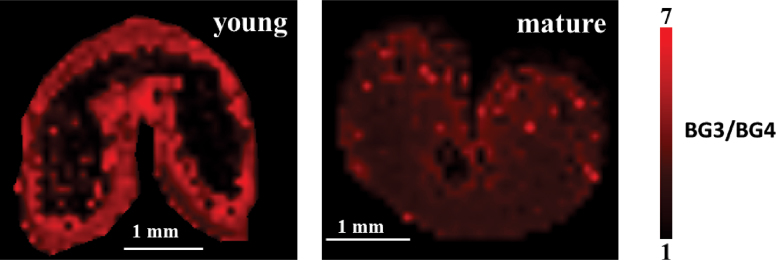
Intensity of the BG3 signal normalized to the BG4 signal in young and mature wheat. BG, β-glucan.

Some differences in the BG composition could be detected between mature and young wheat grains through imaging of the ratio of cellobiosyl-glucose (BG3) to cellotriosyl-glucose (BG4) (Fig. 4). A significantly higher amount of BG3 than BG4 was observed in both developmental stages, revealing a specific composition of wheat BG compared with other crops, as reported previously (Wood, 2002; Lazaridou and Biliaderis, 2007). Consistently with the results displayed in Fig. 3, it is also clear that, in young grains, BG are primarily distributed in the outer layers.

**Fig. 5.**
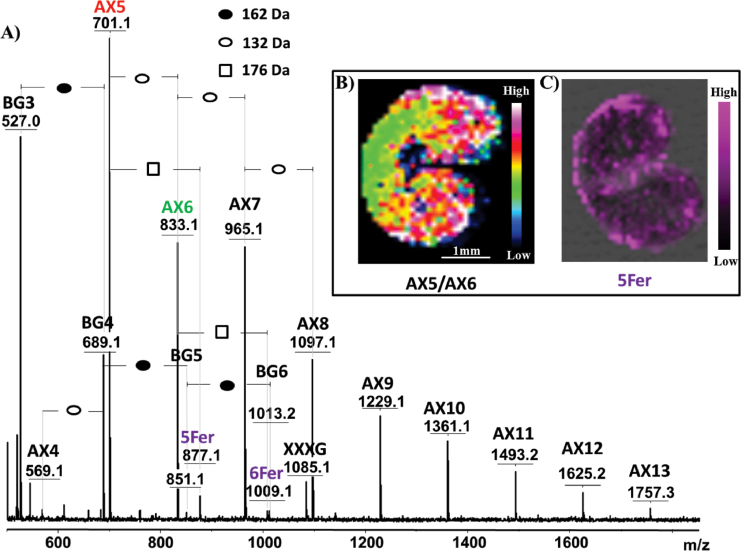
Localization of specific ions in mature wheat. (A) Average MALDI mass spectrum of mature wheat sections following *in situ* enzymic hydrolysis using lichenase and xylanase. (B) Intensity of ions at 701 m/z (AX5) normalized to the intensity at 833 m/z (AX6). (C) Spatial distribution of ions at 877 m/z (assigned to feruloylated AX5, 5Fer). The numbers after BG or AX indicate the degree of polymerization of the oligomers. When feruloylation is observed, numbers before Fer indicate the degree of polymerization of AX, which carries feruloylation (Fer). AX, arabinoxylan oligomer; BG, β-glucan oligomer; XXXG, xyloglucan heptasaccharide, used as internal standard.

Subtle variations in the spatial distribution of AXOS depending on the degree of arabinosyl substitution could be detected through MALDI MSI. Fig. 5 displays the results obtained from the MALDI imaging analysis of mature wheat endosperm following *in situ* enzymic hydrolysis using lichenase and xylanase, and Fig. 5B shows a plot of the AX5/AX6 distribution gradient across the endosperm. Considering the action of xylanase (Saulnier *et al.*, 2009), it can be deduced that DP5 AXOS (AX5) is a mono-substituted xylotetraose, with a single l-arabinofuranosyl residue at position 3 of the second xylopyranosyl residue from the nonreducing end (XA^3^XX; AXOS nomenclature according to Fauré *et al.*, 2009), while DP6 AXOS (AX6) is di-substituted with l-arabinofuranosyl residue, at both positions 2 and 3 of the same xylopyranosyl residue (XA^2+3^XX), as it is presented in Fig. 1. Fig. 5B shows that AX5 was more concentrated in the peripheral tissues of the grain, suggesting that the degree of AX substitution was lower in these layers than in starchy endosperm cell walls, consistent with the previous results of Philippe *et al.* (2006*c*).

The detected peaks shown in Fig. 5A include peaks corresponding to feruloylated AXOS (masses shifted by 176Da). Mapping of these feruloylated ions showed that these molecules are primarily located in the outer layer of mature wheat (Fig. 5C). Notably, due to different ionization efficiencies for feruloylated and nonferuloylated species, it was not possible to deduce the level of arabinose esterified through ferulic acid from these data, as feruloylated species are expected to display a higher sensitivity to laser beam irradiation due to their benzyl group.

**Fig. 6.**
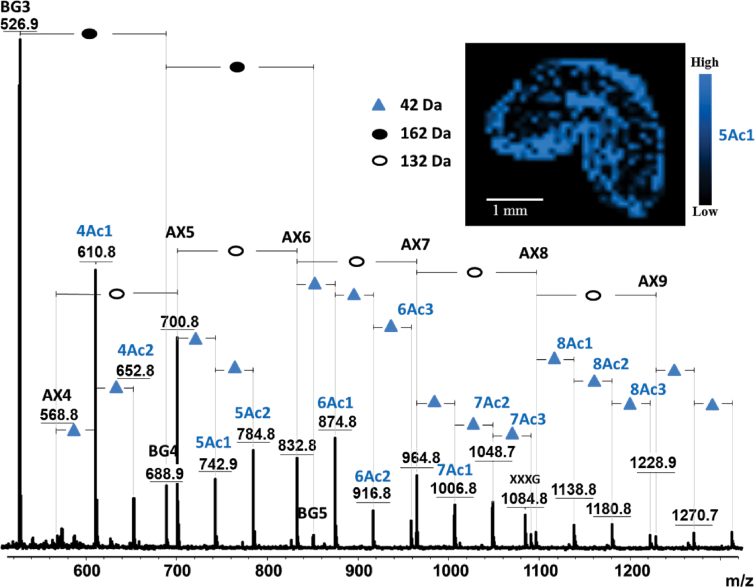
Average mass spectrum of young wheat sections following *in situ* enzymic digestion using lichenase and xylanase. The insert shows the distribution of the ions at 743 m/z (mono-acetylated AX5, 5Ac1) across the tissue section. AX, arabinoxylan oligomer; BG, β-glucan oligomer; numbers after BG and AX indicate the degree of polymerization of the oligomers; Ac indicates the presence of acetylation on AX; numbers before Ac indicate the degree of polymerization of AX acetylation; numbers after Ac indicate the degree of acetylation at the corresponding AX oligomer; XXXG, xyloglucan heptasaccharide, used as internal standard.

**Fig. 7.**
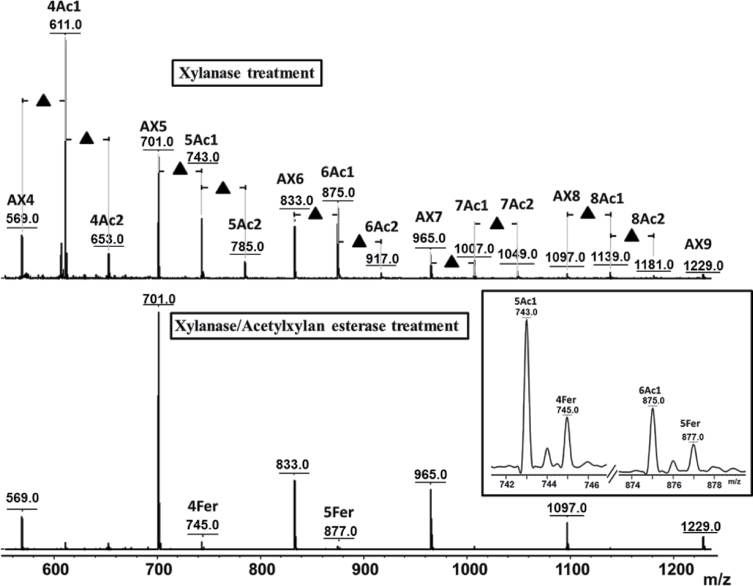
Average MALDI mass spectra of young wheat section following xylanase treatment (A) and coupled xylanase/acetylxylan esterase treatment (B). The insert in Fig. 7B provides an enlarged region of the MS spectrum showing the presence of feruloylated AX4 and feruloylated AX5 at 745 and 877 m/z, respectively. AX, arabinoxylan oligomer; BG, β-glucan oligomer; Ac indicates the presence of acetylation on AX oligomers; Fer indicates the presence of feruloylation; numbers before Ac indicate the degree of polymerization of AX acetylation; numbers after Ac indicate the degree of acetylation at the corresponding AX oligomer. The same nomenclature is used for feruloylated AX.

The above finding emphasizes a unique feature of MALDI MSI compared to other imaging techniques, in that fine structural details, including chemical changes, can be directly deduced from the obtained mass spectra, together with the locations of molecules. Another illustration is given in Fig. 6, in which AX and BG oligomers released following the *in situ* digestion of a young wheat grain is displayed. A 42-Da shift of intense ions from the AXOS series can be observed, suggesting the existence of abundant acetylation of AX in young endosperm, particularly in the outer layer of the tissue. Several (up to three) acetyl groups were detected depending on the degree of AXOS polymerization. Biely *et al.* (1985) reported observing multiple acetylations of xylans after xylanase treatment. However, until recently, the acetylation of AX in the early stages of wheat development had not been reported, as far as is known. Thus, the results obtained in the present study needed to be confirmed through further experiments and observations. First, 42-Da adducts were specifically observed on AXOS in young grains and not on GOS in the same grain (Fig. 6) or on AXOS in mature grains (Fig. 5). The application of a DHB/aniline matrix, rather than a DHB/DMA matrix was performed to assess the carbohydrate origins of the peaks assigned to acetylated AXOS derivatives. This analysis resulted in a 75-Da mass shift (i.e. a mass shift from 93 to 18Da) for all AXOS-related peaks, due to the formation of a Schiff base between the reducing end of the AXOS and the amine moiety of the matrix molecule (Snovida *et al.*, 2006) (Supplementary Fig. S4 available at *JXB* online). This result showed that the 42-Da-shifted series of peaks presented in Fig. 6 reflected acetylated AXOS. Further direct evidence was obtained after subjecting the samples to treatment with an acetylxylan esterase. As shown in Fig. 7, this treatment led to the complete disappearance of the 42-Da-shifted peaks, revealing that these peaks originated from acetylation at the xylan backbone.

Interestingly, Fig. 7B also shows that following the acetylxylan esterase treatment, some peaks assigned to feruloylated AXOS became visible. These species were likely always present but were masked by the acetylated AXOS peaks (the masses of the feruloylated peaks coincide with the second isotope of the acetylated species). Compared to Fig. 5, a higher relative intensity (10-fold) between the feruloylated and nonferuloylated signals was observed in young versus mature wheat (0.007 and 0.06, respectively). This observation confirms that feruloylation increases during maturation, consistent with the increased quantity of AX observed as well as previous reports (Philippe *et al.*, 2006*c*, 2007; Toole *et al.*, 2009).

## Discussion

Although the composition and spatial distribution of AX and BG in diverse regions of wheat endosperm at various stages of maturation have been described using different microscopy and immunolabelling techniques, some questions remain concerning the fine structural variability of these polymers across the grain and/or upon maturation. This study exploited the potential of mass spectrometry as an imaging technique to provide fine molecular and tissue localization information at a resolution of ~100 μm. This method, which was first introduced in the late 1990s, has been used in other studies to analyse metabolites, lipids, peptides or small proteins. Here, this work adapted this technique to investigate the structural variation of cell-wall polysaccharides in wheat endosperm during maturation.

First, the method optimization strategy addressed *in situ* enzymic degradation. As far as is known, this study represents the first attempt to apply polysaccharide-hydrolytic enzymes to intact tissues to monitor the spatial variation of the chemical structures of cell-wall polysaccharides across plant tissues. Obel *et al.* (2009) described the *in situ* enzymic digestion of xyloglucans in *Arabidopsis* seedlings prior to MALDI analysis, but this experimental approach resulted in significant diffusion of the compounds. Following the approach presented here, efficient hydrolysis was achieved compared to the enzymic fingerprinting methodology previously applied to flour (Dervilly *et al.*, 2000), grain (Saulnier *et al.*, 2009) or isolated cell-wall polysaccharides (Sørensen *et al.*, 2007). Although diffusion of the oligosaccharides *in situ* could not be completely excluded, it was maintained below the resolution of the images (~100 μm), thereby providing comprehensive maps where the locations and structures of molecules can be reliably observed simultaneously. A second important point concerns the normalization of the signal to enable comparison of tissues (e.g. between different stages of development). Such comparison was achievable through the introduction of a neutral xyloglucan oligosaccharide (XXXG), which is commercially available as a pure material and was further used as an internal standard to normalize all other peak intensities. Note that, although the mass difference between XXXG and the analysed oligomers is significant, the transmission efficiency is likely conserved over this mass range. This is evidenced by the fact that highly accurate quantification of MT (which exhibits the same mass as BG3) has been achieved, thereby validating the use of XXXG as an internal standard. Moreover, this methodological work addressed the choice of the MALDI matrix. Consistent with previous findings regarding the crystallization homogeneity (resulting in little ‘shot-to-shot’ variation) of the matrix and its efficiency in the detection of neutral oligosaccharides (Snovida and Perreault, 2007; Ropartz *et al.*, 2011), the DHB/DMA matrix was shown to be particularly suited for imaging the products released from AX and BG following hydrolysis. The matrix layer was homogeneous, thereby limiting artefactual variations of the signal due to matrix ‘hot spots’. In addition, DHB/DMA provided neat mass spectra, with no signals originating from molecular species other than the investigated compounds. Interference with phenolic compounds or other metabolites present at the surface of the tissue is a critical issue for the application of MSI to plant tissues.

Applying the optimized method to image young and mature wheat endosperm led to successful confirmation of previous findings regarding the quantitative aspects and structural variations of AX and BG polysaccharides upon seed maturation. Consistent with previous results (Philippe *et al.*, 2006*a*), segregation of AX and BG was observed in young seeds, with both polymers preferentially being located in the outer layers of the endosperm (while their distribution was more uniform in mature grains). It must be noted that the spatial resolution in MALDI MSI (~100 μm in this work) is not sufficient to determine which of the external tissues of the endosperm is detected, especially at the mature stage when the pericarp collapses (Supplementary Fig. S5 available at *JXB* online). Nevertheless, some published knowledge about the enzymic degradability of outer tissues suggests that, in the case of mature wheat, the AXOS released at the peripheral tissues of the endosperm (see, for example, Fig. 5) originate not from the pericarp but rather from the aleurone cells or from the intermediate tissues between aleurone cells and pericarp (Supplementary Fig. S5). Indeed, at this stage of development, the pericarp was reported to be resistant to treatment by xylanase (Beaugrand *et al.*, 2004). A later deposition of AX compared to BG was also observed using MALDI MSI, consistent with these published data. Although the physiological relevance has not been clearly established, increased AX deposition during maturation might strengthen the cell-wall architecture, with a more compact polymer network being formed to accommodate the accumulation of starch and proteins (Philippe *et al.*, 2006*a*).

The BG3/BG4 ratio observed in the present study (close to 7 for young grains and more than 4 for mature ones) is consistent with known data on different cereals: the BG3/BG4 ratio reported for mature grains is in the range of 3.0–4.5 for wheat, 1.8–3.5 for barley, 1.9–3.0 for rye, and 1.5–2.3 for oat (Tosh *et al.*, 2004; Lazaridou and Biliaderis, 2007). However, in contrast to the findings of Toole *et al.* (2010), who reported the BG3/BG4 ratio to be stable upon maturation, this work clearly observed a decrease in this ratio from young to mature stages, which suggests a higher contribution of cellotriosyl compared to cellotetraosyl sequences in the BG structures of young seeds. The role of BG in the mechanical and physicochemical properties of cell walls is incompletely understood, and the impact of the structural variations of BG has been the subject of various—and contradictory—hypotheses. It seems established however that BG3/BG4 plays important contribution to chain–chain association and water solubility (Lazaridou and Biliaderis, 2007).

Interestingly, beyond confirmation of well-established knowledge, the unbiased detection achieved by MALDI MS provided new and exciting insights into the structural features of AX and BG polymers upon grain development. In particular, acetylation of AX was clearly demonstrated in young seeds. As far as is known, this important modification of AX has not yet been described in young wheat, because in many studies on young wheat (Obel *et al.*, 2002; Saulnier *et al.*, 2009; Toole *et al.*, 2010), the applied analytical conditions (an alkaline medium) have resulted in a loss of acetic acid (Sørensen *et al.*, 2007). Other studies (Philippe *et al.*, 2006*a*; Robert *et al.*, 2011) have employed immunolabelling methods, and due to the specificity of the designed antibodies, they could therefore have failed to detect the acetylated forms of AX. More surprisingly, acetylation has not been observed via Fourier transform-infrared microscopy (Philippe *et al.*, 2006*b*; Saulnier *et al.*, 2009; Toole *et al.*, 2010), Raman (Philippe *et al.*, 2006*c*; Toole *et al.*, 2009), or nuclear magnetic resonance (Toole *et al.*, 2009, 2010) analyses. The current work did not detect significant acetylation in the mature endosperm, despite the fact that up to 4% of the xylose released from the cell wall is reportedly acetylated (Rhodes *et al.*, 2002; Sørensen *et al.*, 2007). The level of acetylation in native wheat grain endosperm is potentially too low to be successfully detected using MALDI MSI. Indeed, Sørensen *et al.* (2007) showed that the signal intensity of acetylated AXOS is weak compared to the signal of feruloylated AXOS, which were detectable in the present study, but at a low intensity (Figs 5 and 7). Sørensen *et al.* (2007) and Rhodes *et al.* (2002) demonstrated the acetylation of AX indirectly by titrating the acetic acid produced following alkaline treatment. Furthermore, Rhodes *et al.* (2002) used cell-wall material isolated from aleurone tissue. The origin of the cell-wall material used in the study by Sørensen *et al.* (2007) was not well defined, but it was also potentially enriched in aleurone tissue. The low level of acetylation observed previously might confirm the results of the present study, in which acetylated AXOS could not be detected on native grains (i.e. without enrichment in the aleurone tissues).

The biological function of acetyl substituents in the cell wall is not well understood (Gille *et al.*, 2011), but it is plausible that the increased acetylation of AX in young wheat could make the cell walls more extensible, as acetyl groups might hinder the stacking of polymer chains (Bacon *et al.*, 1975; Kabel *et al.*, 2007). This would support the less rigid cell-wall architecture due to weaker polymer interactions during the young stages of development. In addition, this result provides insight into the substrate specificity of *T. viride* endo-1,4-β-xylanase, as the identification of acetylated AXOS suggests that acetyl residues did not impede the action of this enzyme. Endoxylanases are classified into different glycosyl hydrolase (GH) families, in the CAZy database, among which GH10 and GH11 xylanases hydrolyse AX (Berrin and Juge, 2008). *T. viride* endo-1,4-β-xylanase is a GH11 xylanase that preferentially digests the unsubstituted regions of the AX backbone. In contrast, GH10 xylanases, which are less hampered by the presence of 4-*O*-methyl-d-glucuronate, acetate, and α-l-arabinofuranosyl substituents along the xylan backbone, cleave within decorated regions (Berrin and Juge, 2008). Thus, it is generally accepted that acetylation makes xylan more resistant to xylanase treatment (Biely *et al.*, 1986; Zhang *et al.*, 2011), and the hydrolysis of xylan can be enhanced through the removal of acetyl groups, as shown in hardwood xylans (Zhang *et al.*, 2011). As suggested by the present results and the findings of previous studies (Biely *et al.*, 1997; Sørensen *et al.*, 2007), it seems that xylanases from both family 10 and 11 tolerate the presence of small acetyl substituents on the main xylan chain.

Furthermore, feruloylated AXOS were observed in mature wheat after xylanase digestion and in young wheat following the cooperative action of xylanase and acetylxylan esterase. The preferential mapping of these structures to the outer layer of the tissue is consistent with previous findings showing that the ferulic acid content is much higher in the aleurone layer than in other endosperm regions (Philippe *et al.*, 2006*c*, 2007). Feruloyl groups have not been found to impede the action of endo-1,4-β-xylanase (Lequart *et al.*, 1999; Vardakou *et al.*, 2003; Sørensen *et al.*, 2007). In young grains, feruloylated species were only detected following combined treatment with xylanase and acetylxylan esterase, as the detection of feruloylated AXOS was likely masked in the presence of more prominent acetylated species, generating isotopes of the same masses. The results obtained in the present study showed that, while the ratio of AXOS remains constant during maturation, these molecules show a greater degree of feruloylation in mature grains. Although it has been reported that there is one ferulic acid molecule per 28 arabinose units in mature wheat (Sørensen *et al.*, 2007), it is difficult to quantify the actual ratio of feruloylated/nonferuloylated AXOS by MSI due to the different ionization efficiencies of these compounds. Acting as a cross-linking agent between polymeric chains or between polysaccharides and lignin, ferulic acid contributes to cell-wall assembly, promoting tissue cohesion and restricting cell expansion (Saulnier *et al.*, 2007*b*). It is therefore reasonable to propose that the proportion of feruloylated AX increases in mature wheat tissues.

In conclusion, the applied MALDI MSI approach is proven to be a valuable method for investigating the compositional and structural variations of AX and BG upon the maturation of wheat endosperm. The sensitivity and ease-of-use of the MSI approach make this strategy particularly attractive. A tremendous advantage of this methodology lies in the specificity and accuracy of the structural information provided through MS analysis and the comprehensiveness of the method. This approach led to the identification of endogenous acetylation of AX in young seeds for the first time. This study proposes that MALDI MSI will receive wide interest and provide many new insights in the field of plant polysaccharide segregation and structural characterization. With slight adaptations, for instance concerning the enzyme used or pretreatment of plant tissues to enhance enzyme action (as for lignocellulosic material), the described method can be easily expanded to other tissues and/or other polysaccharides in various plant samples.

## Supplementary material

Supplementary data are available at *JXB* online.

Supplementary Fig. S1. Structure of xyloglucan heptasaccharide (XXXG) used as internal standard in MALDI MSI experiments.

Supplementary Fig. S2. MALDI MS spectra obtained following in-tissue and in-solution digestion of mature wheat grain with xylanase and lichenase.

Supplementary Fig. S3. Maltotriose response over a concentration range of 1 to 14 μg mm^–2^, using (A) 0.2 μg mm^–2^ XXXG and (B) 0.02 μg mm^–2^ XXXG (xyloglucan heptasaccharide) as internal standard. Each spot was replicated three times.

Supplementary Fig. S4. MALDI average mass spectra of AX released following *in situ* digestion of young wheat tissue using xylanase, obtained with DMA/DHB matrix and aniline/DHB matrix.

Supplementary Fig. S5. Autofluorescence of young and mature wheat grain cross-sections.

Supplementary Data
