# Supplementary material for: New insights into the structural and spatial variability of cell-wall polysaccharides during wheat grain development, as revealed through MALDI mass spectrometry imaging
Source: J Exp Bot. 2014 Mar 5;65(8):2079–91. doi: 10.1093/jxb/eru065 (PMC3991742; doi:10.1093/jxb/eru065)
Supplement: Supplementary Data [file supp_65_8_2079__index.html]

New insights into the structural and spatial variability of cell-wall polysaccharides during wheat grain development, as revealed through MALDI mass spectrometry imaging — New insights into the structural and spatial variability of cell-wall polysaccharides during wheat grain development, as revealed through MALDI mass spectrometry imaging — Supplementary Data 

# New insights into the structural and spatial variability of cell-wall polysaccharides during wheat grain development, as revealed through MALDI mass spectrometry imaging

## Supplementary Data

Data files

**Files in this Data Supplement:**

- Supplementary Data - Supplementary Data
