# Supplementary material for: New insights into the structural and spatial variability of cell-wall polysaccharides during wheat grain development, as revealed through MALDI mass spectrometry imaging
Source: J Exp Bot. 2014 Mar 5;65(8):2079–91. doi: 10.1093/jxb/eru065 (PMC3991742; doi:10.1093/jxb/eru065)

Supplemental Figure S1. Structure of xyloglucan heptasaccharide (XXXG) used as internal standard in MALDI MSI experiments.

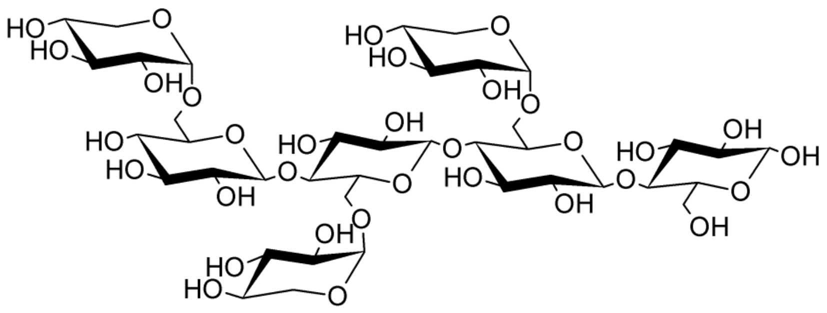

Supplementary Figure S2. MALDI MS spectra obtained following (A) in-tissue and (B) in- solution digestion of mature wheat grain with xylanase and lichenase. Nomenclature: BG-beta glucan oligomer; AX-arabinoxylan oligomer; the number after BG (3-6) or AX (4-13) indicates the degree of polymerization of oligomers; the number before Fer (5, 6) indicates the degree of polymerization of AX, which carries feruloylation (Fer);XXXG-xyloglucan heptasaccharide used as internal standard.

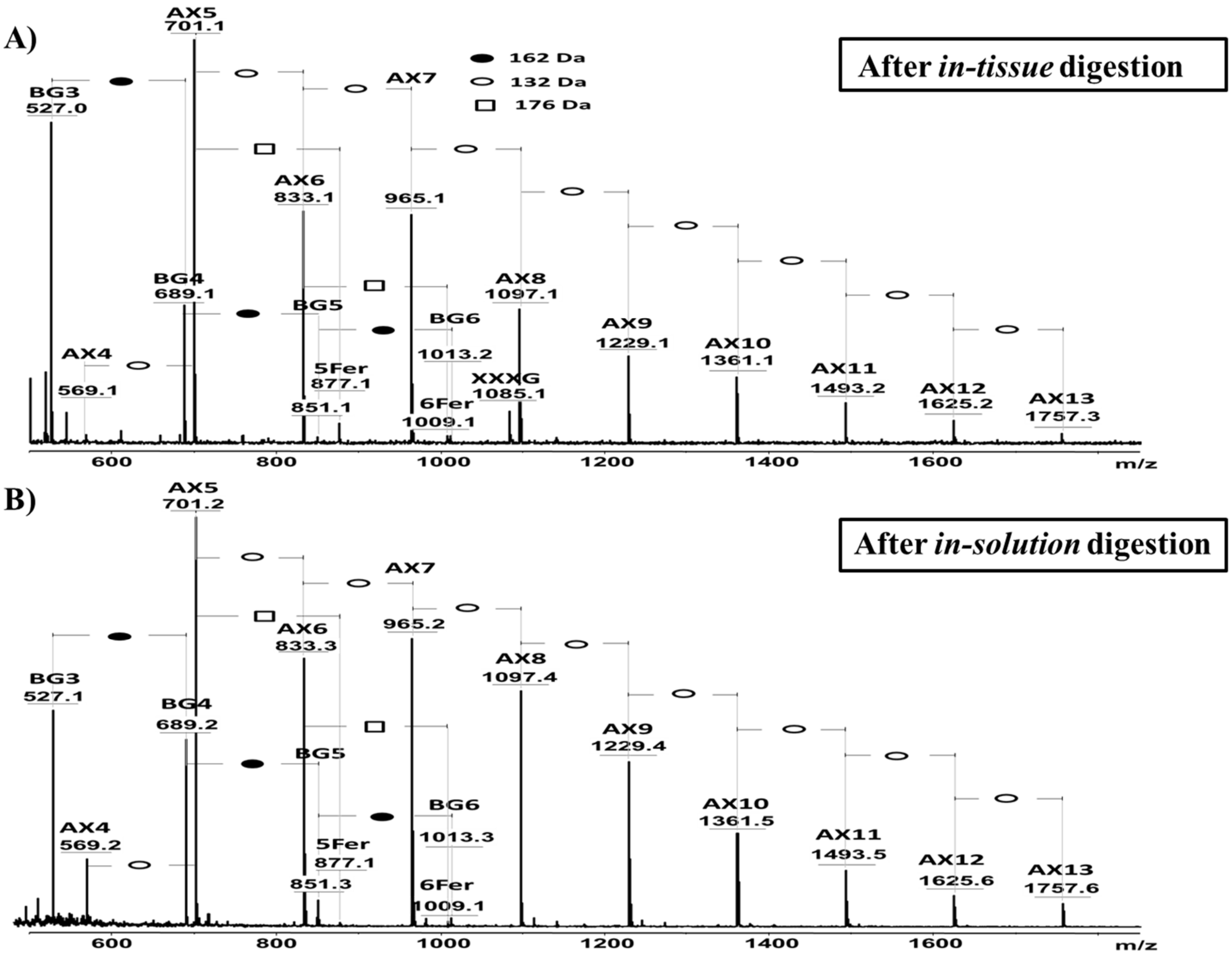

Supplemental Figure S3. Maltotriose (MT) response over a concentration range of 1 to 14 µg mm-2, using (A) 0.2µg mm-2 XXXG and (B) 0.02µg mm-2 XXXG (xyloglucan heptasaccharide) as internal standard. Each spot was replicated three times.

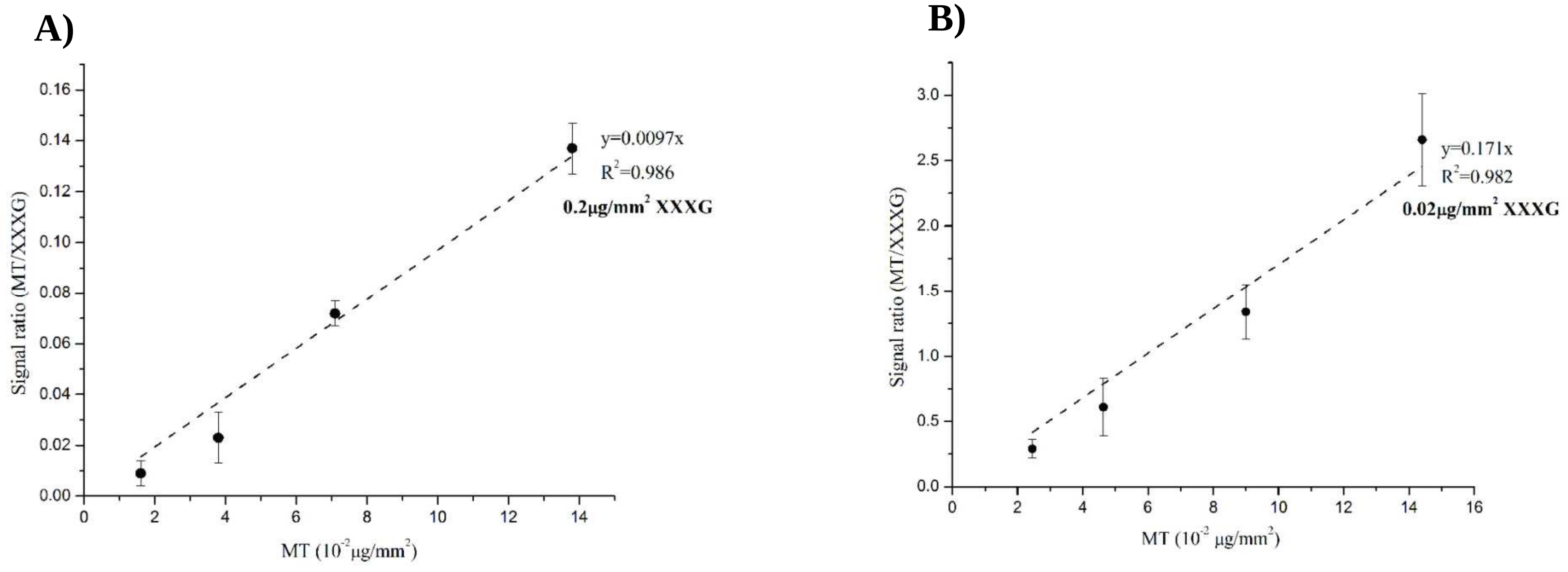

Supplemental Figure S4. MALDI average mass spectrum of AX released following in situ digestion of young wheat tissue using xylanase, obtained with (A) DMA/DHB matrix and (B) aniline/DHB matrix. Nomenclature: number after AX (5, 6, 7) gives the degree of polymerization of AX; Ac indicates presence of acetylation on AX; number before Ac (5, 6, 7) indicates the degree of polymerization of acetylated AX species, while the number after Ac (1, 2) indicates the degree of acetylation. Stars indicate 75-Da shifts corresponding to Schiff base products.

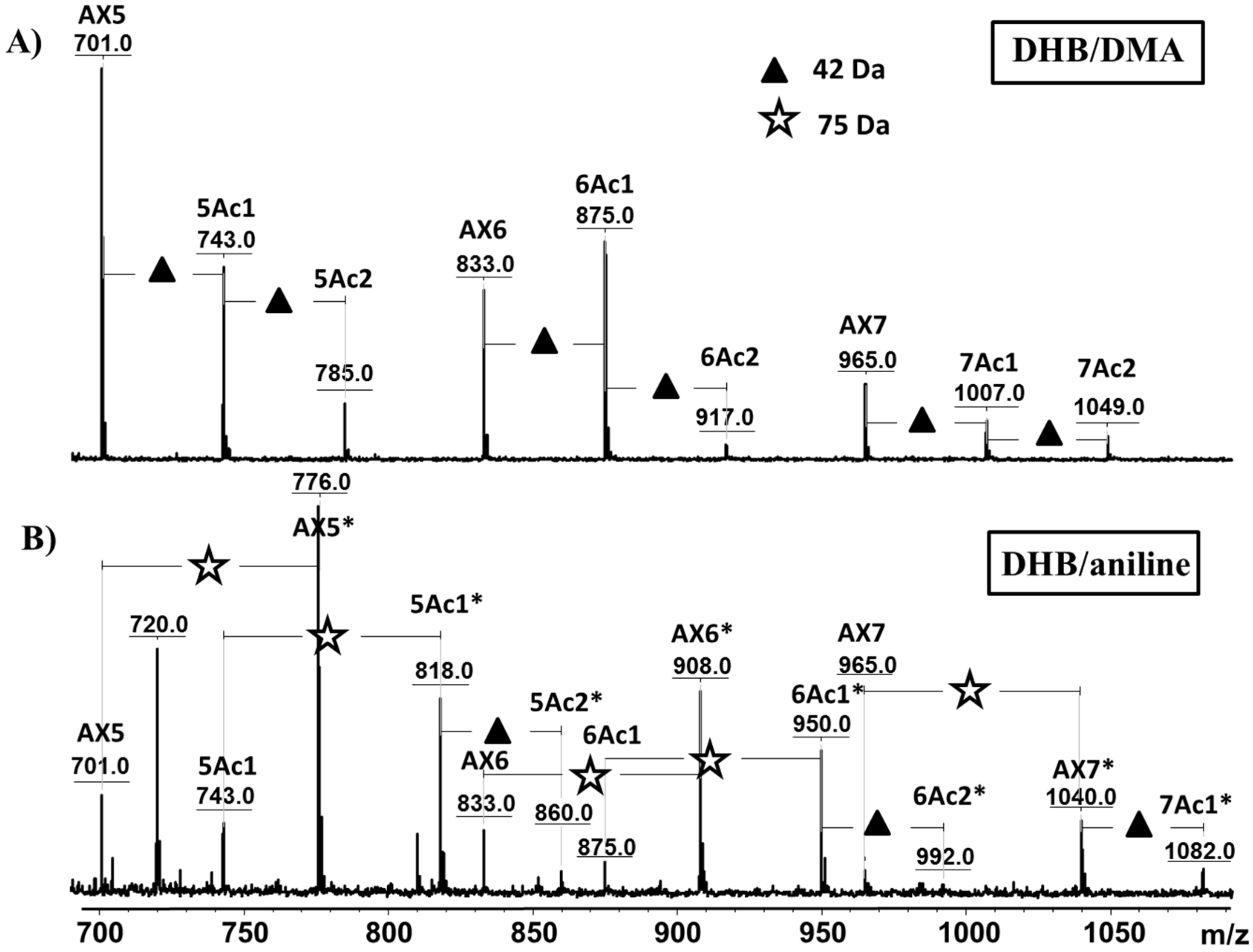

Supplementary Figure S5. Autofluorescence of (A) young and (C) mature wheat grain cross-section observed on a LEICA DMRD microscope, with a band pass filter at 340-380nm used as excitation filter and a fluorescence signal detected at 425nm. The sections marked with squares in (A) and (C) are enlarged in (B) and (D) panels, respectively, so detailed organization of wheat layers is visible. Nomenclature: op- outer pericarp; crc- cross cells; sc- seed coat; ne- nucellar epidermis; al-aleurone layer; pc-prismatic cells; cc-central cells. Note that the MALDI MSI pixel size does not allow concluding whether the signal detected at the peripheral tissues of the grain originates from the outer pericarp, the aleurone layer, or from intermediate layers.

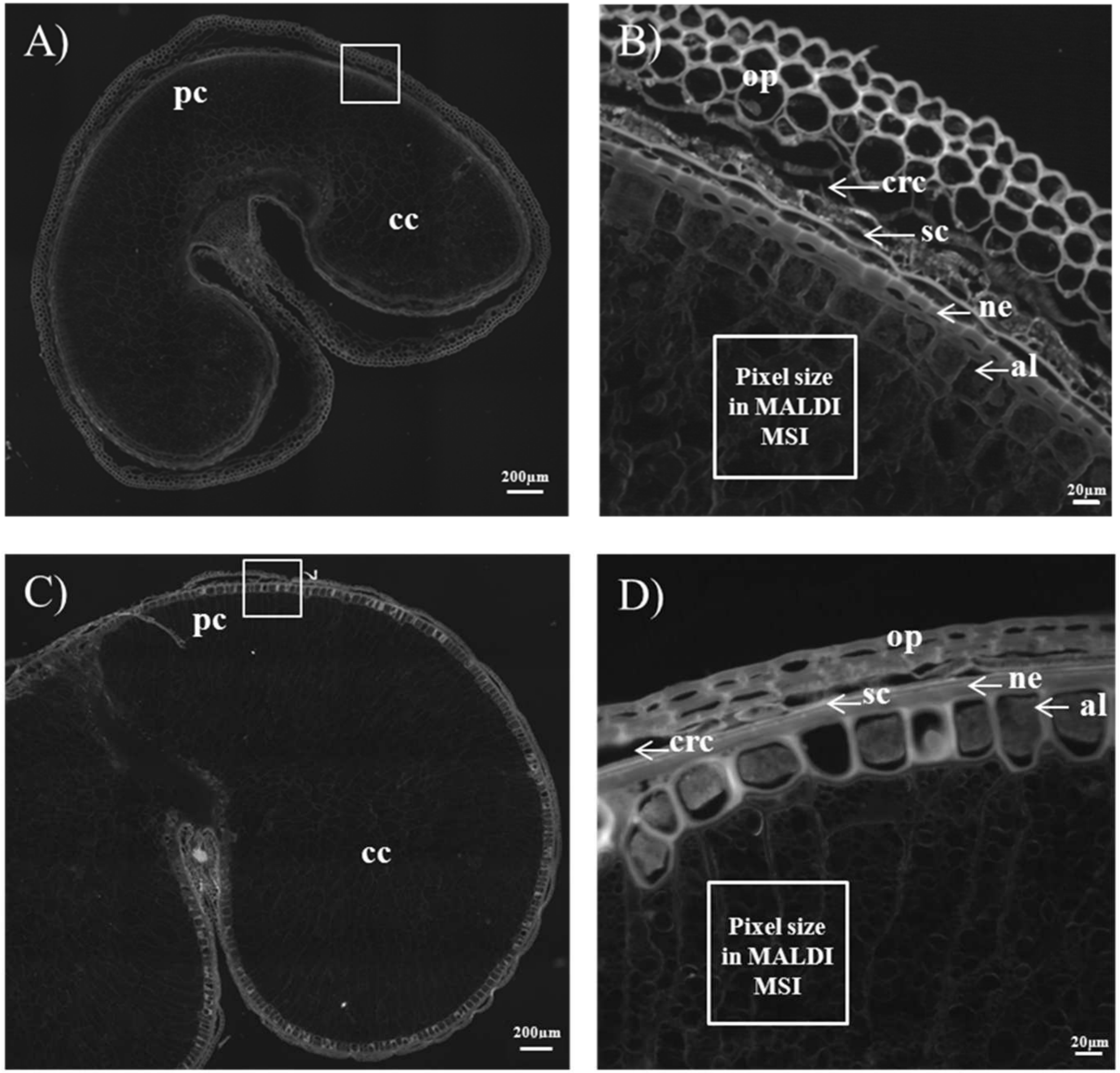

Supplement: Supplementary Data [file supp_eru065_jexbot113324_file001.pdf]
